# Mammary cell proliferation and catabolism of adipose tissues in nutrition-restricted lactating sows were associated with extracellular high glutamate levels

**DOI:** 10.1186/s40104-018-0293-6

**Published:** 2018-11-05

**Authors:** Heju Zhong, Peng Wang, Yumo Song, Xiaoling Zhang, Lianqiang Che, Bin Feng, Yan Lin, Shengyu Xu, Jian Li, De Wu, Qiaofeng Wu, Zhengfeng Fang

**Affiliations:** 1 Key Laboratory for Animal Disease Resistance Nutrition of the Ministry of Education, Animal Nutrition Institute, Sichuan Agricultural University, Chengdu, 611130 China; 2 Acupuncture and Moxibustion College, Chengdu University of Traditional Chinese Medicine, Chengdu, 611137 China

**Keywords:** Glutamate, Insulin sensitivity, Lipolysis, Mammary cell proliferation

## Abstract

**Background:**

Persistent lactation, as the result of mammary cellular anabolism and secreting function, is dependent on substantial mobilization or catabolism of body reserves under nutritional deficiency. However, little is known about the biochemical mechanisms for nutrition-restricted lactating animals to simultaneously maintain the anabolism of mammary cells while catabolism of body reserves. In present study, lactating sows with restricted feed allowance (RFA) (*n* = 6), 24% feed restriction compared with the control (CON) group (*n* = 6), were used as the nutrition-restricted model. Microdialysis and mammary venous cannulas methods were used to monitor postprandial dynamic changes of metabolites in adipose and mammary tissues.

**Results:**

At lactation d 28, the RFA group showed higher (*P* < 0.05) loss of body weight and backfat than the CON group. Compared with the CON group, the adipose tissue of the RFA group had higher (*P* < 0.05) extracellular glutamate and insulin levels, increased (*P* < 0.05) lipolysis related genes (*HSL* and *ATGL)* expression, and decreased (*P* < 0.05) glucose transport and metabolism related genes (*VAMP8*, *PKLR* and *LDHB*) expression. These results indicated that under nutritional restriction, reduced insulin-mediated glucose uptake and metabolism and increased lipolysis in adipose tissues was related to extracellular high glutamate concentration. As for mammary glands, compared with the CON group, the RFA group had up-regulated (*P* < 0.05) expression of Notch signaling ligand (*DLL3*) and receptors (*NOTCH2* and *NOTCH4*), higher (*P* < 0.05) extracellular glutamate concentration, while expression of cell proliferation related genes and concentrations of most metabolites in mammary veins were not different (*P* > 0.05) between groups. Accordingly, piglet performance and milk yield did not differ (*P* > 0.05) between groups. It would appear that activation of Notch signaling and adequate supply of glutamate might assist mammogenesis.

**Conclusions:**

Mammary cell proliferation and catabolism of adipose tissues in nutrition-restricted lactating sows were associated with extracellular high glutamate levels.

**Electronic supplementary material:**

The online version of this article (10.1186/s40104-018-0293-6) contains supplementary material, which is available to authorized users.

## Background

For lactating animals, the prerequisite for high milk production is to have adequate nutrition intake and high mobilization of body reserves [1]. Modern high prolific sows tend to have lower feed intake, especially under conditions of heat stress during lactation [2]. Decreased feed intake reduces nutrient supplementation and causes nutritional deficiencies. In nutritional deficiency status, maternal reserves, such as lipids from adipose tissues, is mobilized to offer energy and substrates to mammary glands to satisfy lactation [3]. Hormone-sensitive lipase (HSL) is involved in triglyceride decomposition of white adipose tissue, and *HSL* gene silencing leads to enhanced insulin-stimulated glucose uptake and de novo lipogenesis. In contrast, insulin resistance expedites the lipolytic rate of white adipose tissue [4]. It appears that insulin and HSL work together to regulate the catabolism and anabolism of adipose tissues.

Well-developed mammary glands is essential for secretion of milk [5], and persistent lactation is dependent on mammary cell proliferation [6], a key biological process of mammary gland development [7]. It has been illustrated that nucleotides are essential substrates for the synthesis of nucleic acids, and the amount of intracellular de novo nucleotides defines the rate of cell proliferation [8–10]. Moreover, Notch signaling is shown to play a key role in the regulation of nucleotide metabolism [11] and mammary cell proliferation [12].

Although the mechanisms that regulate lipolysis of adipose tissues and proliferation of mammary cells were previously studied separately, few study reports are available on the biological explanation of the simultaneous occurrence of body reserves mobilization and mammary cells proliferation in vivo during lactation. Proliferating mammary epithelial cells need glutamate to participate in nucleotide metabolism [13], non-essential amino acids synthesis and tricarboxylic acid (TCA) cycle [14]. However, in adipocytes, glutamate can downregulate insulin-mediated glucose uptake and Akt signaling [15]. Therefore, we hypothesized that proliferation of mammary cells and catabolism of adipose tissues in nutrition-restricted lactating sows were associated with glutamate metabolism. To test this hypothesis, primiparous lactating sows with restricted feed allowance were used as the mild nutritional restriction model. Microdialysis methods as previously described [16] were used to simultaneously monitor the dynamic changes of key metabolic markers in extracellular fluids of subcutaneous adipose tissues and mammary glands.

## Methods

### Animals and diets

The protocol of this study was approved by the Animal Care and Use Committee of Animal Nutrition Institute, Sichuan Agricultural University. A total of 12 lactating crossbred (Landrace × Yorkshire) primiparous sows were used in this experiment from lactation d 0 to 28, and the day of farrowing is defined as lactation d 0. Sows and their litter were individually housed in fixed farrowing crates, and heat lamps provided supplemental heat to the pigs. Within 48 h of farrowing, all litters were standardized to have 10 piglets, and were weighed to ensure that each litter weight is uniform. Sows were allocated (*n* = 6 per group) to the control (CON) and restricted feed allowance (RFA) based on body weight at farrowing. Sows were fed a corn and soybean meal-based diet during lactation (Table 1), and this diet was formulated based on nutrient requirements of lactating sows [17]. All lactating sows were fed 0 kg at lactation d 0, fed 2 kg at lactation d 1, from lactation d 2 to 7 the feed allowance increased by 0.5 kg/d. Then, the CON group sows were fed 5 kg/d from lactation d 8 to 27, while the RFA group sows were only fed 3.8 kg/d referred to previous studies [18, 19]. According to our previous study [20], the feed allowance of 5 kg/d within a group was to minimize the variation of potential body reserves mobilization and milk yielding performance among animals [21]. Lactating sows and suckling piglets had free access to water throughout the experimental period, meanwhile, there was no creep feed offered to suckling piglets.

**Table 1:** Ingredients and composition of diet

Ingredient, %		Composition^a^	
Corn	56.92	ME, Mcal/kg	3.15
Soybean meal	28.04	DM, g/kg	879.2
Wheat bran	4.89	CP, g/kg	200.6
Fish meal	3.77	EE, g/kg	63.5
Soybean oil	3.50	CF, g/kg	21.8
Salt	0.25	SID Lys, g/kg	9.4
Sodium Bicarbonate	0.20	SID Met, g/kg	2.9
Limestone	0.67	SID Met+Cys, g/kg	5.3
Dicalcium phosphate	0.45	SID Thr, g/kg	6.2
Vitamin premix^b^	0.07	SID Trp, g/kg	2.0
Mineral premix^3^	1.00	Calcium, g/kg	6.8
Choline chloride (50%)	0.24	STTD phosphorus, g/kg	3.2
Total	100		

^a^*ME* metabolizable energy, *DM* dry matter, *CP* crude protein, *EE* ether extract (crude fat), *CF* crude fiber, *SID* standardized ileal digestible, *Lys* lysine, *Met* methionine, *Cys* cysteine, *Thr* threonine, *Trp* tryptophan, *STTD* standardized total tract digestible

^b^Provided the following per kilogram of diet: vitamin A, 24,500 IU; vitamin D_3_, 7,000 IU; vitamin E, 52.5 IU; vitamin K, 7 mg; *D*-biotin, 0.28 mg; folic acid, 3.5 mg; niacin, 70 mg; *D*-pantothenic acid, 35 mg; vitamin B_2_, 17.5 mg; vitamin B_1_, 7 mg; vitamin B_6_, 10.5 mg; vitamin B_12_, 0.07 mg

^c^Provided the following per kilogram of diet: copper, 24 mg; iron, 90 mg; manganese, 31 mg; zinc, 119 mg; selenium, 0.18 mg; iodine, 0.17 mg

### Growth performance determination

Body weight of each sow was weighed after overnight fasting at lactation d 0 and 28. And backfat of sows was measured on lactation d 0 and 28 by ultrasound (Renco Lean-Meater, USA). The backfat was measured 3 times on each right and left side of the sow, 65 mm from the last (12^th^) backbone (P2 point). Sows’ backfat is represented as mean values from six measurements. Feed intake of each sow was recorded daily. Piglets were weighed individually on d 0, 7, 14, 21 and 28.

### Ear venous blood collection

At lactation d 0 and 28, following an overnight period of feed withdrawal, blood samples (10 mL) from each sow were withdrawn from ear vein into heparinized tubes, and were put on ice before centrifugation, then immediately centrifuged for 10 min at 2,550×*g* and 4 °C. The supernates were divided into some subsamples and stored at − 20 °C until they were analyzed.

### Mammary gland venous catheter surgery

With an overnight fasting, the lactating sow was weaned and transferred from the farrowing unit to the surgery room at lactation d 28. And Mammary venous cannulation procedure referred to a previous research [22]. The sow was restrained and injected with 2 mL (1 mL:0.5 mg) atropine sulfate injection (Taiji Group Southwest Pharmaceutical Co. Ltd., China), and anesthetized with 2 mL Shumianning (Nanjing Agricultural University, China) through the distal end of an ear vein 15 min latter. Surgical anesthesia was maintained using sevoflurane (Lunan Pharmaceutical, China) given to effect (2%) via a closed-circuit gas anesthesia. The sow was placed in left lateral recumbency and the hair of anterior mammary glands and above the shoulder was clipped. The skin was scrubbed using standard surgical procedures. An incision was made approximately 7.5 to 8 cm above the nipple between the first and the second gland, parallel to the ventral border of the fold [22]. The fat and the connective tissue were dissected to find the main mammary vein, and it was created an opening through the fascia by eye scissors. A 110-cm heparinized cannula (Tygon Tubing 1.27 mm i.d. × 2.29 mm o.d., Component Supply Co. Fort Myers, FL, USA), prepared according to previous references [22, 23], filled with 0.1% heparin sodium (Sigma-Aldrich, USA) solution, was inserted into mammary vein slowly for a distance of 8 cm. At this position, the first cuff blocked further entry. The cannula was fixed in position by suturing the cuffs horizontally to the connective tissue bed underlying the vein. The distal end of the catheter was passed subcutaneously from the site of incision to the dorsal midline between shoulders. This was done by attaching the catheter to a puncture needle. The distal end of cannula was adapted for sampling by inserting 1 cm long blunt 18 gauge needle fixed to a heparin cap.

### Tissue collection

After the mammary gland venous catheter surgery, about 2 cm^3^ mammary gland parenchyma tissue was collected from the left anterior third mammary glands using surgical methods. And about 2 cm^3^ subcutaneous adipose tissue was thoroughly collected at the right P2 point using surgical methods. Then, tissues thoroughly flushed with ice-cold sterile saline to remove blood and dry with filter paper, and frozen in liquid nitrogen for subsequent total RNA isolation.

### Microdialysis and mammary venous blood collection

After 3 h of recuperation, sows had fully recovered their vitality. After that, the lactating sow was immobilized in a special cage with hammock after mammary gland catheter surgery. Lactating sows could stand, lies and ingest freely in this cage. To collect the extracellular fluid of living mammary glands and subcutaneous adipose tissues, microdialysis procedure was executed as previously described [24]. In brief, prior to insertion of the 24-mm (14-mm shaft and 10-mm membrane) microdialysis probe (CMA 20; CMA Microdialysis AB, Sweden), 0.5 mL lidocaine (10 mg/mL) was administrated subcutaneously at the target tissue. Microdialysis probe was inserted via a split tubing and introducer (CMA Microdialysis AB, Sweden), and specific operation referred to CMA 20 Elite Microdialysis Probe manual (CMA Microdialysis AB, Sweden). One microdialysis probe was placed in the bottom of the left third mammary gland and directed towards the nipple. Another microdialysis probe was placed in abdominal subcutaneous adipose tissue distance from 20 cm above the left side third nipple. Both of them were perfused with hydroxyethyl starch 130/0.4 and sodium chloride injection (Chongqing Daxin Pharmaceutical Co., Ltd., China), which was pumped at a speed of 2 μL/min with a CMA 4004 Syringe Pump (CMA Microdialysis AB, Sweden). After 1 h of equilibration, samples were collected, the sampling frequency was 15 min and collection time sustained for 150 min. Samples were collected by CMA 470 refrigerated fraction collector (CMA Microdialysis AB, Sweden). The outgoing perfusate was stored at − 80 °C for subsequent analysis. Meanwhile, the sows were not fed in the first 15 min and were provided 0.5 kg feed (sow could consume it within 15 min) at the start of 16^th^ min after sample collection. And 10 mL mammary venous blood was taken into heparinized tubes per 15 min. In each case, the first 3 mL of fluid withdrawn was discarded, subsequent 10 mL of withdrawals were considered to be representative blood samples. After that, the cannula was flushed with 0.1% heparin sodium (Sigma-Aldrich, USA) solution. The mammary gland venous blood was centrifuged at 2,550×*g* for 10 min and 4 °C, and the plasma was stored at − 20 °C.

### Calculation

The milk yield of sows was estimated from Hansen et al. [25]. The calculation of milk yield was based on litter weight gain and litter size.

### Plasma metabolites analysis

Frozen plasma samples at lactation d 0 and 28 were thawed at 4 °C, the creatinine in ear venous plasma was determined by 7020 automatic analyzer (Hitachi, Japan). And the creatinine assay kit (Maccura Biotechnology Co., Ltd., China) was used in this machine. The urea, total protein, glucose, triglyceride, NEFA and total cholesterol in the mammary gland venous blood were also determined by 7020 automatic analyzer (Hitachi, Japan). And the NEFA assay kit (Beijing Strong Biotechnologies, Inc., China) and other relevant assay kits (Maccura Biotechnology Co., Ltd., China) were used in this machine.

### Microdialysis samples analysis

Extracellular fluid samples were thawed at 4 °C until they became liquid, they were analyzed with an ISCUS microdialysis analyzer (CMA Microdialysis AB, Sweden), a chemistry analyzer using enzymatic reagents and colorimetric measurements. Substrate-specific reagents (CMA Microdialysis AB, Sweden) for glucose, lactate, pyruvate, and glutamate were used when the samples were being analyzed. The subcutaneous adipose tissue fluid in in microdialysis equilibration tube was used to represent adipose tissue in fasting state. Adipose tissue insulin level in equilibration tube was tested by Porcine Insulin ELISA Assay Kit (Nanjing Jiancheng Bioengineering Institute, China).

### RNA extraction and real-time qPCR

RNA extraction and real-time qPCR was performed as previously described [21]. Briefly, before RNA isolation, the adipose tissue and mammary gland were grinded in liquid nitrogen, and total RNA was isolated using RNAiso Plus regent (Takara, Japan). The concentration and purity of RNA were determined by using a NanoDrop 2000 (Thermo Scientific, USA), the range of OD_260_:OD_280_ between 1.8 and 2.0 was acceptable. The RNA integrity was verified by agarose gel electrophoresis. cDNA was generated using the PrimeScript RT reagent Kit with gDNA Eraser (Takara, Japan). Real-time qPCR was performed on an ABI 7900HT Sequence Detection System or QuantStudio 6 Flex Real-Time PCR System (Life Technologies, USA) with SYBR PREMIX EX TAQ II (Takara, Japan), and a melting curve analysis was also carried out. The thermal cycling parameters were as follows: 95 °C for 30 s, followed by 40 cycles at 95 °C for 5 s and 60 °C for 34 s, followed by 95 °C for 15 s, 60 °C for 1 min and 95 °C for 15 s. Relative mRNA abundances of the determined genes in the adipose tissue and mammary gland samples were calculated by using the 2^-∆∆CT^ method [26]. All expression data were normalized to endogenous control gene both TATA boxbinding protein (*TBP*) and β-actin (*ACTB*) expression [27]. Each data was then normalized to control group within an experiment. Primers were designed by company (Sangon Biotech (Shanghai) Co., Ltd., China) and Primer-BLAST was done at NCBI (https://www.ncbi.nlm.nih.gov/). Information about primer pairs for selected genes was summarized in Additional file 1 [see Additional file 1].

### Statistical analysis

Data are presented as least-squares means with pooled SEM, unless otherwise specified. The pen was considered the experimental unit for statistical analysis. Data were analyzed by using the GLM procedure of SAS 9.4 (SAS Institute). The least significant difference test was used to compare the group means when the F test in the analysis of variance table was significant. However, if these data did not show a normal distribution or homogeneous variance, the rank-sum test was used, and data were analyzed by using the NPAR1WAY procedure of SAS 9.4 (SAS Institute). As previously described [28], the repeated measures data of metabolites in mammary gland vein and the extracellular fluid of adipose tissue and mammary glands, respectively, were analyzed by using the MIXED procedure of SAS 9.4 (SAS Institute), and the best appropriate covariance structure was selected and used in REPEATED Statement. The differences were considered significant at *P* < 0.05.

## Results

### Performance of sows and piglets

The CON group sows had higher (*P* < 0.05) feed intake, lesser (*P* < 0.05) body weight and backfat loss than the RFA group sows at lactation d 28 (Table 2). However, the body weight and average daily gain of piglets (Table 2) and milk yield of sows were not significantly different between the two groups.

**Table 2:** The performance of lactating sows and piglets

Item	Treatment^a^		Pooled SEM	P*
	CON	RFA		
Sows				
Feed intake, kg/d	4.84	3.59	0.15	< 0.01
Body weight, kg				
d 0	199.20	198.05	6.21	0.86
d 28	184.08	160.43	7.81	0.014
Body weight loss, kg				
d 0–28	15.87	37.62	5.48	< 0.01
Backfat, mm				
d 0	15.25	16.08	1.73	0.91
d 28	12.40	11.25	1.75	0.53
Backfat loss, mm				
d 0–28	2.75	4.83	0.81	0.03
Milk yield, kg/d				
d 0	3.78	3.73	0.54	0.93
d 7	7.83	7.90	0.21	0.76
d 14	9.17	9.20	0.56	0.96
d 21	9.39	9.30	0.65	0.90
d 28	9.10	8.88	0.66	0.76
Piglets				
Body weight, kg				
d 0	1.31	1.30	0.01	0.93
d 7	2.48	2.59	0.11	0.37
d 14	3.81	3.59	0.27	0.87
d 21	5.11	4.69	0.48	0.63
d 28	6.63	6.37	0.60	0.67
Average daily gain, kg				
d 0–7	0.17	0.18	0.02	0.40
d 7–14	0.19	0.14	0.03	0.10
d 14–21	0.19	0.16	0.04	0.44
d 21–28	0.22	0.22	0.02	0.87

^a^*CON* control, *RFA* restricted feed allowance

^*^It was considered significant at *P* < 0.05

### Plasma creatinine concentration and adipose tissue insulin level

**Fig. 1:**
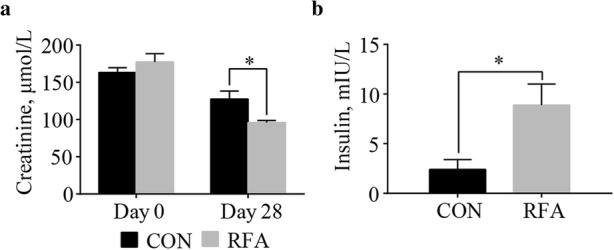
The creatinine contents in plasma and insulin concentrations in adipose tissues in fasting state. In fasting state, creatinine levels (**a**) in ear venous plasma at lactation d 0 and 28, and insulin contents in adipose tissue fluid (**b**) at lactation d 28. Values are least-squares means with SE. Mean values were significantly different from those of the control group: *P < 0.05. It was considered significant at *P* < 0.05. CON, control; RFA, restricted feed allowance

At lactation d 28, the fasting levels of plasma creatinine in ear vein (Fig. 1a) was lower (*P* < 0.05) in the RFA than in the CON group. The fasting level of insulin (Fig. 1b) in the extracellular fluid of subcutaneous adipose tissues was higher (*P* < 0.05) in the RFA than in the CON group.

### Dynamic changes of key metabolites in extracellular fluid of subcutaneous adipose tissues

**Fig. 2:**
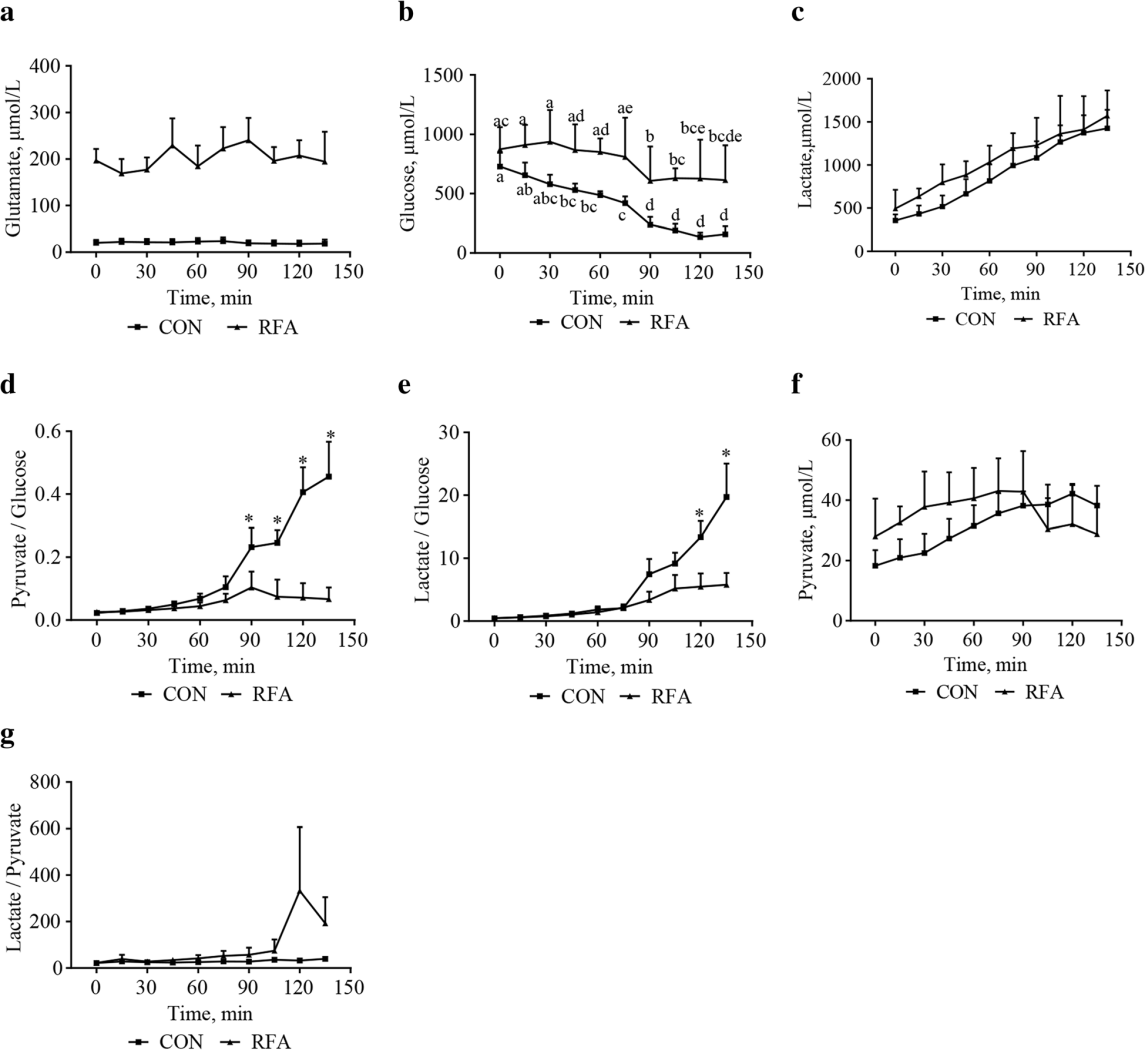
Metabolites concentrations in extracellular fluids of subcutaneous adipose tissues after ingestion at lactation d 28. Zero min represents the extracellular fluid collected within 15 min before ingestion, representing a fasting state. This figure shows effects of diet, time, and diet × time interactions on dynamic changes of glutamate (**a**, *P*_diet_ = 0.001, *P*_time_ = 0.51, *P*_diet × time_ = 0.43), glucose (**b**, *P*_diet_ = 0.18, *P*_time_ = 0.001, *P*_diet × time_ = 0.76), lactate (**c**, *P*_diet_ = 0.59, *P*_time_ < 0.0001, *P*_diet × time_ = 0.97), Pyruvate/Glucose (**d**, *P*_diet_ = 0.029, *P*_time_ < 0.0001, *P*_diet × time_ = 0.001), Lactate/Glucose (**e**, *P*_diet_ = 0.20, *P*_time_ < 0.0001, *P*_diet × time_ = 0.002), pyruvate (**f**, *P*_diet_ = 0.75, *P*_time_ = 0.22, *P*_diet × time_ = 0.72), Lactate/Pyruvate (**g**, *P*_diet_ = 0.23, *P*_time_ = 0.45, *P*_diet × time_ = 0.49). Values are least-squares means with SE. The differences between time points within a group were indicated by superscripts with no common letters, while differences between groups at a specific time point were indicated by asterisk. It was considered significant at *P* < 0.05. CON, control; RFA, restricted feed allowance; Pyruvate/Glucose, the ratio of pyruvate to glucose; Lactate/Glucose, the ratio of lactate to glucose; Lactate/Pyruvate, the ratio of lactate to pyruvate

Extracellular glutamate concentrations in the RFA group were significantly higher (*P* < 0.05) than that in the CON group (Fig. 2a). After a meal, extracellular glucose concentrations of adipocytes decreased, with the first significant (*P* < 0.05) decrease observed at 45 min postprandial in the CON group while 90 min postprandial in the RFA group. Gradually increased (*P* < 0.0001) lactate concentrations (Fig. 2c) were observed in extracellular fluids of adipocytes in both of groups. Compared with the CON group, the RFA group showed decreased (*P* < 0.05) ratio of pyruvate to glucose (pyruvate/glucose, Fig. 2d) at 90 min postprandial, and lower (*P* < 0.05) ratio of lactate to glucose (lactate/glucose, Fig. 2e) at 120 min and 135 min postprandial, respectively.

### Dynamic changes of key metabolites in extracellular fluids of mammary tissues

**Fig. 3:**
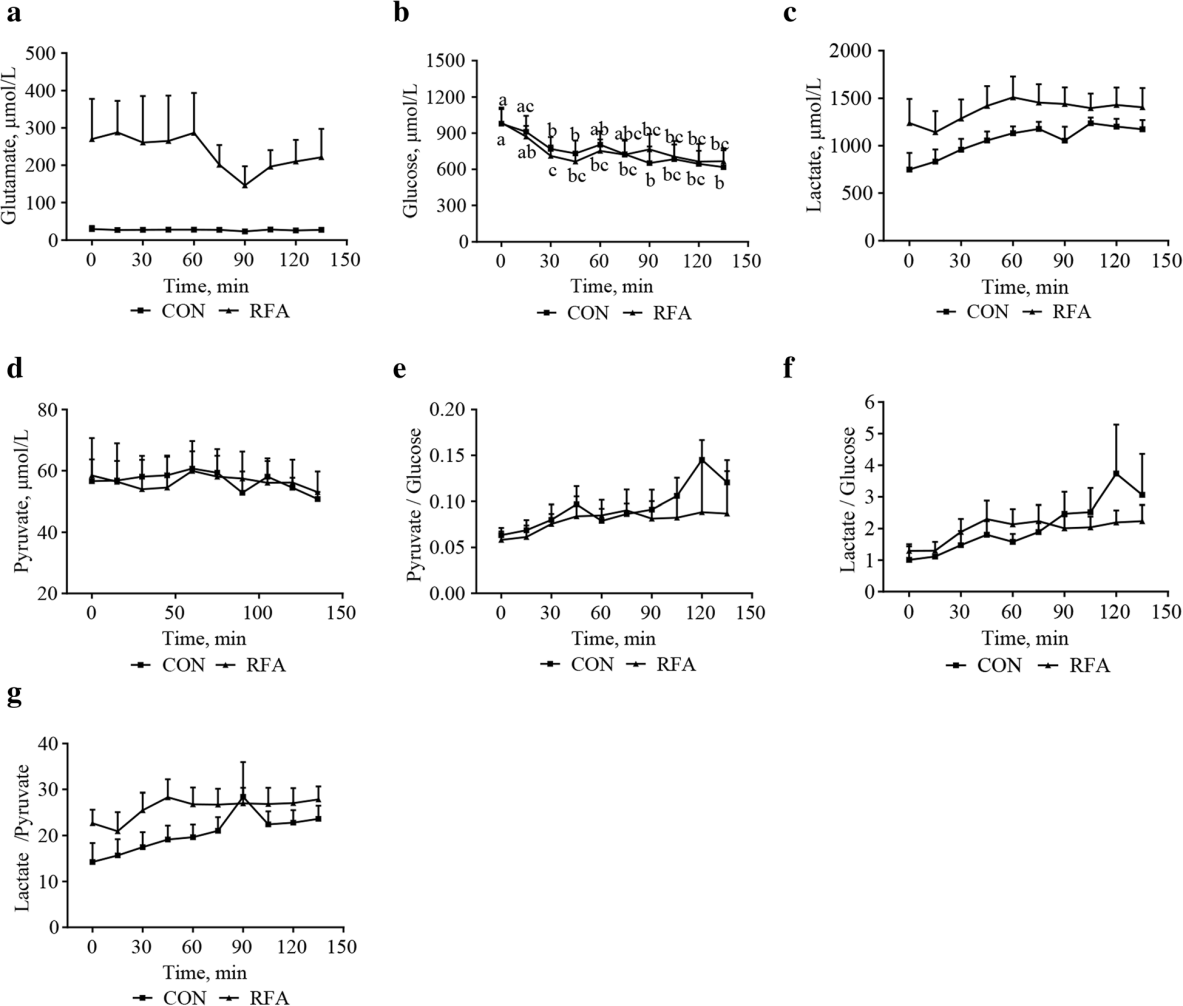
Metabolites concentrations in mammary extracellular fluids after ingestion at lactation d 28. Zero min represents the extracellular fluid collected within 15 min before ingestion, representing a fasting state. This figure shows effects of diet, time, and diet × time interactions on dynamic changes of glutamate (**a**, *P*_diet_ = 0.006, *P*_time_ = 0.96, *P*_diet × time_ = 0.98), glucose (**b**, *P*_diet_ = 0.99, *P*_time_ = 0.005, *P*_diet × time_ = 0.94), lactate (**c**, *P*_diet_ = 0.25, *P*_time_ = 0.06, *P*_diet × time_ = 0.42), pyruvate (**d**, *P*_diet_ = 0.99, *P*_time_ = 0.11, *P*_diet × time_ = 0.43), Pyruvate/Glucose (**e**, *P*_diet_ = 0.60, *P*_time_ = 0.10, *P*_diet × time_ = 0.62), Lactate/Glucose (**f**, *P*_diet_ = 0.89, *P*_time_ = 0.032, *P*_diet × time_ = 0.36), Lactate/Pyruvate (**g**, *P*_diet_ = 0.22, *P*_time_ < 0.0001, *P*_diet × time_ = 0.26). Values are least-squares means with SE. Differences between groups at a specific time point were indicated by asterisk. It was considered significant at *P* < 0.05. CON, control; RFA, restricted feed allowance; Lactate/Pyruvate, the ratio of lactate to pyruvate; Lactate/Glucose, the ratio of lactate to glucose

Extracellular concentrations of glutamate (*P* = 0.006, Fig. 3a) were higher (*P* < 0.05) in the RFA than in the CON groups, while other metabolites did not differ between groups at each of timepoints evaluated.

### Dynamic changes of key metabolites in mammary veins

**Fig. 4:**
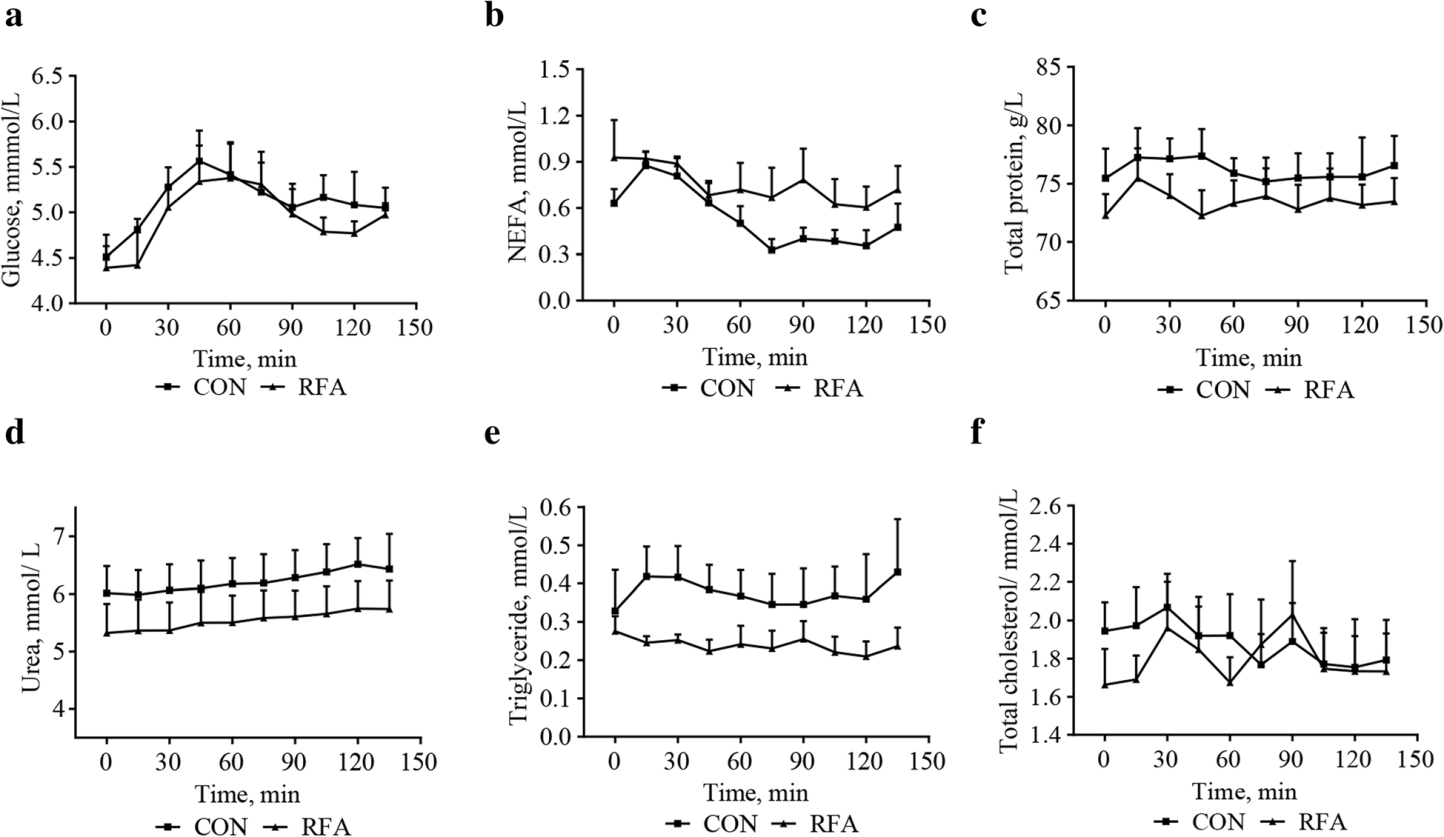
Dynamic changes of metabolites in mammary veins after ingestion at lactation d 28. Zero min represents the blood collected within 15 min before ingestion, representing a fasting state. This Figure shows effects of diet, time, and diet × time interactions on dynamic changes of glucose (**a**, *P*_diet_ = 0.55, *P*_time_ < 0.0001, *P*_diet × time_ = 0.65), NEFA (**b**, *P*_diet_ = 0.20, *P*_time_ = 0.035, *P*_diet × time_ = 0.72), total protein (**c**, *P*_diet_ = 0.38, *P*_time_ = 0.025, *P*_diet × time_ = 0.27), urea (**d**, *P*_diet_ = 0.34, *P*_time_ < 0.0001, *P*_diet × time_ = 0.95), triglyceride (**e**, *P*_diet_ = 0.20, *P*_time_ = 0.51, *P*_diet × time_ = 0.36), total cholesterol (**f**, *P*_diet_ = 0.75, *P*_time_ = 0.09, *P*_diet × time_ = 0.40). Values are least-squares means with SE. It was considered significant at *P* < 0.05. CON, control; RFA, restricted feed allowance; NEFA, nonesterified fatty acid

The concentrations of most metabolites (Fig. 4) were significantly (*P* < 0.05) affected by time, whereas not affected (*P* > 0.05) by diet or diet×time interactions.

### Gene expression in tissues of sows at lactation d 28

**Fig. 5:**
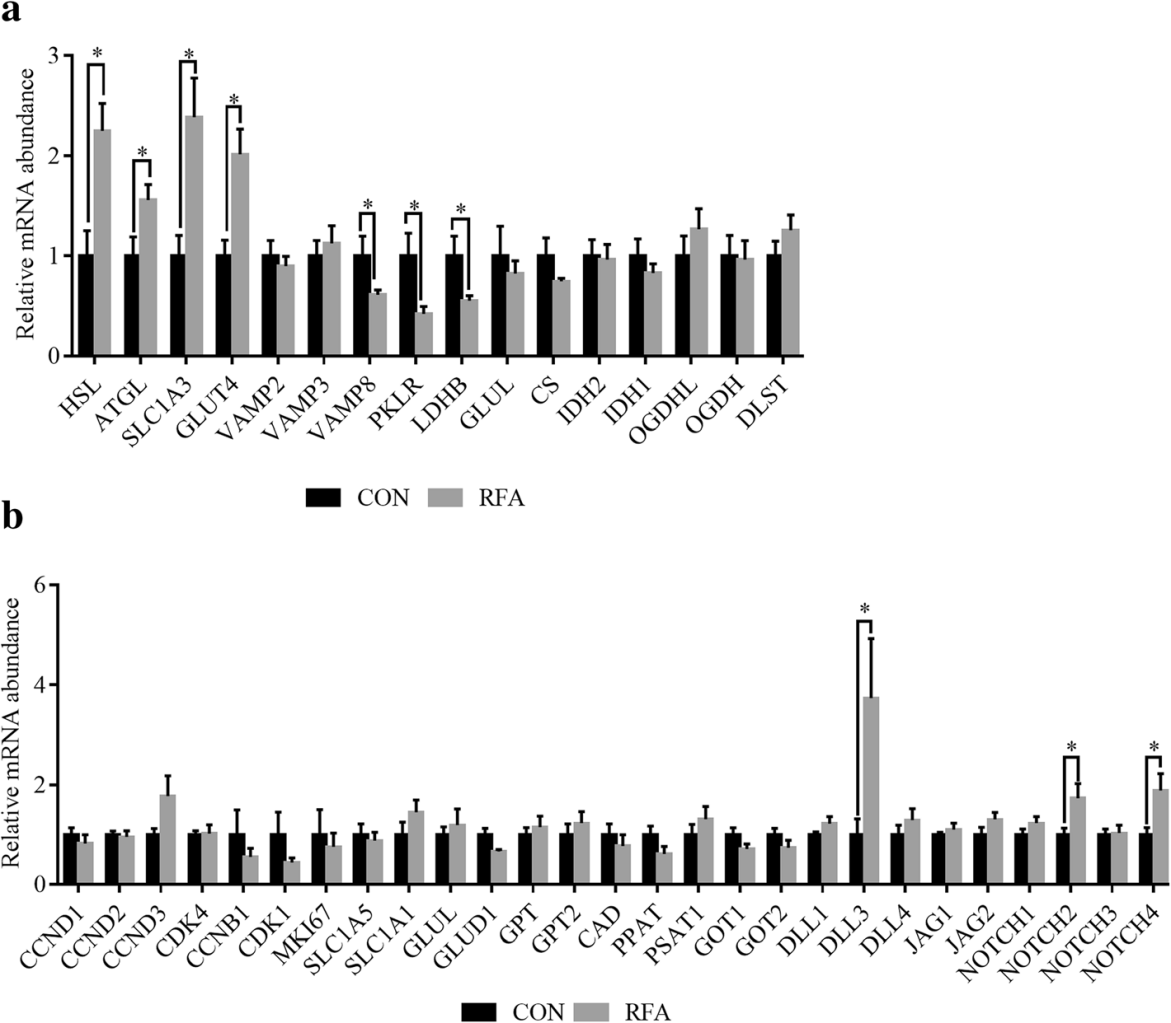
Gene expression in tissues of sows at lactation d 28. In adipose tissue, the relative mRNA abundances of lipolysis (*HSL*, *ATGL*), glutamate and glucose metabolism (*SLCIA3*, *GLUT4*, *VAMP2*, *VAMP3*, *VAMP8*, *PKLR*, *LDHB*, *GLUL*) and tricarboxylic acid cycle (*CS*, *IDH2*, *IDH1*, *OGDHL*, *OGDH*, *DLST*) (**a**). In mammary gland, the mRNA abundances of cell proliferation (*CCND1*, *CCND2*, *CCND3*, *CDK4*, *CCNB1*, *CDK1*, *MKI67*), glutamate and nucleotide metabolism (*SLC1A5*, *SLC1A1*, *GLUL*, *GLUD1*, *GPT*, *GPT2*, *CAD*, *PPAT*, *PSAT1*, *GOT1* and *GOT2*), and Notch signaling pathway (*DLL1*, *DLL3*, *DLL4*, *JAG1*, *JAG2*, *NOTCH1*, *NOTCH2*, *NOTCH3*, *NOTCH4*) (**b**). Values are least-squares means with SE. Mean values were significantly different from those of the control group: *P < 0.05. It was considered significant at *P* < 0.05. CON, control; RFA, restricted feed allowance

In adipose tissues, the mRNA abundance of *HSL*, adipose triglyceride lipase (*ATGL)*, glutamate transporter *SLCIA3* and glucose transporter 4 (*GLUT4*) was higher (*P* < 0.05) in the RFA than in the CON group (Fig. 5a). The mRNA abundance of glutamine synthetase (*GLUL*) and TCA cycle related enzymes (*CS*, *IDH2*, *IDH1*, *OGDHL*, *OGDH* and *DLST*) was not significantly (*P* ≥ 0.05) different between groups (Fig. 5a). However, the mRNA abundance of vesicle associated membrane protein 8 (*VAMP8*), pyruvate kinase (*PKLR*) and lactate dehydrogenase (*LDHB*) was lower (*P* < 0.05) in the RFA than in the CON group (Fig. 5a). In mammary glands, the mRNA abundance of Notch ligand *DLL3*, and Notch receptor *NOTCH2* and *NOTCH4* was higher (*P* < 0.05) in the RFA than in the CON group (Fig. 5b). And the mRNA abundance of cell cycle (*CCND1*, *CCND2*, *CCND3*, *CDK4*, *CCNB1*, *CDK1*), marker of proliferation Ki-67 (*MKI67*), glutamate transporter (*SLC1A5*, *SLCIA1*), *GLUL*, glutamate dehydrogenase (*GLUD1*) and glutamic pyruvictransaminase (*GPT*, *GPT2*) nucleotide synthesis related enzymes (*CAD*, *PPAT*, *PSAT1*, *GOT1* and *GOT2*) was not significantly (*P* ≥ 0.05) different between the RFA and CON groups (Fig. 5b).

## Discussion

In nutritional deficiency status, maternal reserves is mobilized to satisfy the nutrients requirement of mammary glands [29]. On lactation d 28, compared with the CON sows, the RFA sows had higher loss of body weight and backfat and lower plasma creatinine concentration, which is positively correlated with the muscle mass [30]. It appeared that nutritional restriction led to accelerated body reserves loss and negative energy balance.

Given that a majority of energy is stored in adipose tissues, we monitored the change of key metabolic indexes in subcutaneous adipose tissues before and after meal, and determined the expression of some related genes. One important finding is that postprandial glucose concentrations decreased fast in the CON group but slow in the RFA group. There is evidence that the requirement for VAMP 8 in GLUT4 trafficking to the plasma membrane is essential in adipocytes [31], and insulin-stimulated GLUT4 can transport extracellular glucose into cells [32]. In this perspective, although nutritional restriction promoted insulin levels and expression of *GLUT4* in adipose tissues, the relatively lower *VAMP8* expression in adipose tissues of the RFA sows might hinder the GLUT4 trafficking to the plasma membrane and thus impeded the transport of glucose into adipocytes. Furthermore, pyruvate produced by glycolysis enters two different metabolic pathways under the action of pyruvate kinase and lactate dehydrogenase [33]. Compared with the control sows, the RFA sows had lower *PKLR* and *LDHB* expression and lower postprandial pyruvate/glucose and lactate/glucose ratios, illustrating that glucose uptake and its utilization efficiency were reduced in nutritional restriction sows. Glucose uptake by adipose tissues is usually triggered by insulin, but disrupted by insulin resistance, leading to lipolysis [34]. Compared with the CON group, the upregulated expression of *HSL* and *ATGL* in the RFA group contributed to enhanced lipolysis in adipose tissues of the RFA sows [35]. This is in line with more backfat loss in the RFA group than in the CON group. These results indicated that nutritional restriction led to reduced insulin-mediated uptake and utilization of glucose, and increased lipolysis in adipose tissues.

Previous studies suggested that adipose tissues took up more glutamate than other tissues [36], but high glutamate levels could disturb insulin sensitivity by reducing insulin-mediated glucose uptake and phosphorylation of Akt in adipocytes [15]. In present study, it was observed that the extracellular glutamate concentrations in adipose tissues were significantly higher in the RFA group than in the CON group. Meanwhile, compared with the CON group at lactation d 28, the RFA group had higher expression of glutamate transporter *SLC1A3* which can help cells intake glutamate. Therefore, we proposed that high extracellular glutamate levels might lead to lower insulin sensitivity and increased lipolysis of adipose tissues in nutritional restricted sows.

It is established that nutritional restriction usually results in accelerated mobilization of maternal reserves, and nutrients from body reserves partly influx into the mammary glands. The concentrations of most metabolites (glucose, nonesterified fatty acids (NEFA), total protein, urea, triglyceride, total cholesterol) in mammary veins were not different between the CON and RFA groups, suggesting little effect of nutritional restriction by 24% on the transfer of nutrients to the mammary glands.

Persistent lactation is dependent on mammary cell proliferation [7]. In this study, it was found that the expression of cell proliferation related genes (*CCND1*, *CCND2*, *CCND3*, *CDK4*, *CCNB1*, *CDK1*, *MKI67*) in mammary glands was not different between the CON and RFA groups, suggesting the similar status of mammary cell proliferation between groups. In support of this notion, the expression of genes (*CAD*, *PPAT*, *PSAT1*, *GOT1*, *GOT2*) encoding enzymes responsible for the synthesis of nucleotides, the basic materials for DNA synthesis [37], was also not different between the CON and RFA groups. Although most metabolites concentrations in mammary vein showed similar levels between groups, intriguingly, the RFA group had higher extracellular glutamate concentration than the CON group. And the extracellular glutamate concentration did not exceed the maximum transport capacity of the transporter [38]. Thus, the glutamate transport of the RFA group would be increased without altering the transcripts for glutamate transporters relative to the CON group. Glutamate, as the metabolically active amino acid [14], is demonstrated to be the raw materials of the de novo nucleotide [13]. It would appear that the increase in extracellular glutamate concentration in the RFA sows might provide sufficient starting materials for nucleotide synthesis. Otherwise, evidence is available that Notch signaling can sense the nucleotide abundance and regulate the rate of cell proliferation to protect cells from deleterious damage caused by exhausting the nucleotide pool [39]. The RFA group had higher expression of Notch signaling ligand (*DLL3*) and receptors (*NOTCH2*, *NOTCH4*) than the CON group, suggesting activation of Notch signaling in nutrition-restricted sows. Overall, it would appear that under nutritional restriction, the upregulation of Notch signaling pathway was to ensure mammary cell proliferation and nucleotide synthesis, which might promote the accumulation of nucleotide synthesis raw material, glutamate, in mammary glands. The similarity in piglet performance and milk yield between groups provided further evidence for the similar status of mammary cell proliferation between the CON and RFA sows.

Furthermore, the high metabolic activities of milk secreting cells require substantial glutamate via transaminases to couple non-essential amino acid synthesis to α-ketoglutarate generation and tricarboxylic acid cycle anaplerosis [14]. These metabolisms are involved with the proteins encoded by *GLUD1*, *GPT*, *GPT2*, *GOT1* and *GOT2* genes, the expression of which was not different between the CON and RFA groups at lactation d 28. These observations suggested that under nutritional restriction, extracellular high glutamate concentration might favor the metabolism in mammary glands and thus benefit proliferating mammary epithelial cells.

## Conclusions

In conclusion, under nutritional restriction status, high concentration of glutamate in extracellular fluids might be contributory in reducing insulin sensitivity and thus increasing lipolysis of adipose tissues of lactating sows. Moreover, activation of Notch signaling and adequate supply of glutamate in mammary glands might assist mammogenesis.

## Additional file

Additional file 1: Accession number, primer sequence and product size of genes evaluated^1^. (DOCX 24 kb)

## Abbreviations

ACTB: β-actin
ATGL: adipose triglyceride lipase
CF: crude fiber
CON: control
CP: crude protein
Cys: cysteine
DM: dry matter
EE: ether extract (crude fat)
GLUD1: glutamate dehydrogenase
GLUL: glutamine synthetase
*GLUT4*: glucose transporter 4
Lactate/Glucose: the ratios of lactate to glucose
Lactate/Pyruvate: the ratio of lactate to pyruvate
Lactate/Pyruvate: the ratio of lactate to pyruvate
LDHB: lactate dehydrogenase
Lys: lysine
ME: metabolizable energy
Met: methionine
NEFA: nonesterified fatty acid
PKLR: pyruvate kinase
Pyruvate/Glucose: the ratio of pyruvate to glucose
RFA: restricted feed allowance
SID: standardized ileal digestible
STTD: standardized total tract digestible
TBP: TATA boxbinding protein
TCA: tricarboxylic acid
Thr: threonine
Trp: tryptophan
VAMP8: vesicle associated membrane protein 8
