# Supplementary material for: Mammary cell proliferation and catabolism of adipose tissues in nutrition-restricted lactating sows were associated with extracellular high glutamate levels
Source: J Anim Sci Biotechnol. 2018 Nov 5;9:78. doi: 10.1186/s40104-018-0293-6 (PMC6217789; doi:10.1186/s40104-018-0293-6)
Supplement: Supplementary file 1 — Accession number, primer sequence and product size of genes evaluated1. (DOCX 24 kb) [file 40104_2018_293_MOESM1_ESM.docx]

**Additional file 1**

Accession number, primer sequence and product size of genes evaluated^1^

| Name | NCBI Reference Sequence | Primer sequence (5'-3') | Product size, bp |
| --- | --- | --- | --- |
| TBP | XM_021085483.1 |  | 119 |
| Forward |  | GCGATTTGCTGCTGTAATCA |  |
| Reverse |  | TTGCTGCTAGTCTGGACTGTTC |  |
| ACTB | XM_003124280.5 |  | 111 |
| Forward |  | AGAGCAAGAGAGGCATCCTG |  |
| Reverse |  | CACGCAGCTCGTTGTAGAAG |  |
| HSL | NM_214315.3 |  | 126 |
| Forward |  | GCGTGAAGGACAGGACAGTG |  |
| Reverse |  | CCATGGATATGCACGACCAG |  |
| ATGL | NM_001098605.1 |  | 115 |
| Forward |  | GACGTGGAACATCTCGTTCG |  |
| Reverse |  | GTAGATGTGCTTGGCGTTGG |  |
| SLC1A3 | NM_001244425.2 |  | 121 |
| Forward |  | GGATGCGAGCCGTAGTCTAT |  |
| Reverse |  | TTGCCTTCTCTGTGCATGTT |  |
| GLUT4 | NM_001128433.1 |  | 118 |
| Forward |  | AGGCACCCTCACTACCCTCT |  |
| Reverse |  | CAGCATTGCCTTCTTCCTTC |  |
| VAMP2 | XM_003358281.5 |  | 111 |
| Forward |  | TGAACGTGGACAAGGTCCTG |  |
| Reverse |  | TGAGCTTGGCTGCACTTGTT |  |
| VAMP3 | XM_021095270.1 |  | 133 |
| Forward |  | AGCTGGATGACCGAGCAGAT |  |
| Reverse |  | ATGATGACCACGACGCTGAT |  |
| VAMP8 | XM_003354743.4 |  | 128 |
| Forward |  | ACCTGCGGAGTGAAGTGGAG |  |
| Reverse |  | GTGCTCAGATGTGGCTTCCA |  |
| PKLR | XM_021089721.1 |  | 119 |
| Forward |  | CGTGAACCTCCAGAGGACAT |  |
| Reverse |  | CCTGTCACCACAATCACCAG |  |
| LDHB | NM_001113287.1 |  | 110 |
| Forward |  | CAGAGATGGGAACGGACAAT |  |
| Reverse |  | AATTGCCCAGTTGGTGTAGC |  |
| GLUL | NM_213909.1 |  | 125 |
| Forward |  | ATTGTGGAGGCTCACTACCG |  |
| Reverse |  | TCTCCCATGTCGATTCCTTC |  |
| IDH2 | NM_001164007.1 |  | 134 |
| Forward |  | ATGGAACCATCCGGAACATC |  |
| Reverse |  | CTTGTACTGGTCGCCGTGAG |  |
| IDH1 | XM_005672137.3 |  | 125 |
| Forward |  | GGAATTGGACCTGCACAGC |  |
| Reverse |  | TGATGGTGGCACACTTGACA |  |
| CS | NM_214276.1 |  | 130 |
| Forward |  | GGAGCAGGTGTCTTGGCTCT |  |
| Reverse |  | GGCTGTAATGGCTGCACTGA |  |
| OGDHL | XM_021072996.1 |  | 136 |
| Forward |  | GTGAAGTGGCCAGCTCTGTG |  |
| Reverse |  | GAGCCGAAGGCCATGTATTC |  |
| OGDH | XM_003134891.6 |  | 118 |
| Forward |  | CGCTCAAGACCATCATCGAC |  |
| Reverse |  | TGCTCCAGCTCCTTCCTGAT |  |
| DLST | NM_214397.1 |  | 129 |
| Forward |  | TCTGTGCAGGTTCCATCACC |  |
| Reverse |  | CTTGGCCTTAGCAGGAGCAG |  |
| CCND1 | XM_021082686.1 |  | 136 |
| Forward |  | CTTCAAGTGCGTGCAGAAGG |  |
| Reverse |  | CAGGAAGCGGTCCAGGTAGT |  |
| CCND2 | NM_214088.1 |  | 122 |
| Forward |  | GCAGAACTTGCTGACCATCG |  |
| Reverse |  | CACAGACCTCCAGCATCCAG |  |
| CCND3 | NM_001078678.1 |  | 150 |
| Forward |  | TGTCAGGAGCAGATCGAAGC |  |
| Reverse |  | CAGGTGGATGGCTGTGACAT |  |
| CDK4 | NM_001123097.1 |  | 123 |
| Forward |  | CAATGGAGGAGGTGCTGGAG |  |
| Reverse |  | GCAGTGGCACAGACATCCAT |  |
| CCNB1 | NM_001170768.1 |  | 117 |
| Forward |  | TGGTGAATGGACACCAACTC |  |
| Reverse |  | TGCTTTGTAAGCCCTCGATT |  |
| CDK1 | XM_005671013.3 |  | 121 |
| Forward |  | AGTGTGGCCAGAAGTGGAGT |  |
| Reverse |  | TTTCGAGAGCAGATCCAAGC |  |
| MKI67 | XM_013983877.2 |  | 120 |
| Forward |  | AGGGCACAGTCTCTGGAAGA |  |
| Reverse |  | TGCTGGTGTGTTGACTGGTT |  |
| SLC1A5 | XM_003127238.5 |  | 118 |
| Forward |  | TCGATTCGTTCCTGGATCTT |  |
| Reverse |  | ACCTTCACCACAGTGCCATT |  |
| SLC1A1 | NM_001164649.1 |  | 135 |
| Forward |  | CGCTGTGCTGAAGAGAAGAA |  |
| Reverse |  | GTCATTCAACTGCGCGATAA |  |
| GLUL | NM_213909.1 |  | 125 |
| Forward |  | ATTGTGGAGGCTCACTACCG |  |
| Reverse |  | TCTCCCATGTCGATTCCTTC |  |
| GLUD1 | NM_001244501.1 |  | 119 |
| Forward |  | GTCCTGGATTGCTGACACCT |  |
| Reverse |  | CAGTAGCAGAGATGCGTCCA |  |
| GPT | XM_003125440.3 |  | 114 |
| Forward |  | GGATGCCAAGAGAAGAGCAC |  |
| Reverse |  | ATGTACCGAGCCACATCCTC |  |
| GPT2 | XM_013998283.1 |  | 120 |
| Forward |  | CGCCATCCAGGTGAACTATT |  |
| Reverse |  | ATGATGCACAGCACCTTGG |  |
| CAD | XM_021087696.1 |  | 119 |
| Forward |  | CTTGTTCCTCGGAGCCTCAT |  |
| Reverse |  | CTACACTGTCCAGCCGCAAC |  |
| PPAT | XM_003482396.4 |  | 137 |
| Forward |  | ACGCAGATGTGGTGAGCACT |  |
| Reverse |  | CATGTTCGGCTGAATGAAGG |  |
| PSAT1 | XM_021065150.1 |  | 106 |
| Forward |  | ATGAGACCGTGCATGGAGTG |  |
| Reverse |  | GACACATCCACTGGCTTGGA |  |
| GOT1 | NM_213927.1 |  | 117 |
| Forward |  | GGAACCTGGAACCACATCAC |  |
| Reverse |  | CCGACCACTTGGTAGCAGAT |  |
| GOT2 | NM_213928.1 |  | 128 |
| Forward |  | GCCTTCACTGTGGTCTGCAA |  |
| Reverse |  | GTCAGGATGGTCGAGGCAAT |  |
| DLL1 | XM_005659096.3 |  | 124 |
| Forward |  | TGGATTCCTGCACTTCCTCA |  |
| Reverse |  | CAGTCGTCCACGTTGTCCTC |  |
| DLL3 | XM_013988565.2 |  | 103 |
| Forward |  | CGGATGGACCTTGCTTCAAT |  |
| Reverse |  | TCACAGTTGGAGCCTTGGAA |  |
| DLL4 | NM_001244418.1 |  | 108 |
| Forward |  | AGCTCAGCGAGTGTGACAGC |  |
| Reverse |  | CGCAGTGCAGGCCATAATAG |  |
| JAG1 | XM_005672699.3 |  | 107 |
| Forward |  | CAGGCCACGTACACACTGGT |  |
| Reverse |  | GCGCTCTCCAGGTCTCTGTT |  |
| JAG2 | XM_021081545.1 |  | 118 |
| Forward |  | TGGTGAATGGCTACCAGTGC |  |
| Reverse |  | TCAACCAGGTCTTGGCAGAA |  |
| NOTCH1 | XM_021081037.1 |  | 126 |
| Forward |  | GAGGTGAACACGGACGAGTG |  |
| Reverse |  | CACGTCGTACTGGCACAGGT |  |
| NOTCH2 | XM_021090689.1 |  | 120 |
| Forward |  | CGCCAGCCTCCGTATTACTC |  |
| Reverse |  | GTCGGCACAATACTGGCTCA |  |
| NOTCH3 | XM_021083631.1 |  | 117 |
| Forward |  | TTCTGTACGGAGGACGTGGA |  |
| Reverse |  | TCCTGTCCAGCCATTGACAC |  |
| NOTCH4 | NM_001123147.1 |  | 123 |
| Forward |  | CGACCACAATGAGTGCCTGT |  |
| Reverse |  | TCGGTCTCCACCTCACAGAG |  |

^1^Primers were designed by company (SANGON BIOTECH (SHANGHAI) CO., LTD., China) and Primer-BLAST was done at NCBI (https://www.ncbi.nlm.nih.gov/).
